# Female partner experiences of prostate cancer patients’ engagement with a community-based football intervention: a qualitative study

**DOI:** 10.1186/s12889-021-11448-7

**Published:** 2021-07-15

**Authors:** Julie Midtgaard, Tine Tjørnhøj-Thomsen, Mette Rørth, Malene Kronborg, Eik D. Bjerre, John L. Oliffe

**Affiliations:** 1 The University Hospitals Centre for Health Research, Copenhagen University Hospital, Rigshospitalet, Department 9701, Blegdamsvej 9, DK-2100 Copenhagen Ø, Denmark; 2 Department of Clinical Medicine, University of Copenhagen, Blegdamsvej 3B, DK-2200 Copenhagen N, Denmark; 3 Mental Health Centre Glostrup, University of Copenhagen, Nordstjernevej 41, DK-2600 Glostrup, Denmark; 4 National Institute of Public Health, Department of Health and Social Context, University of Southern Denmark, Studiestræde 6, DK-1455 Copenhagen K, Denmark; 5 The University of British Columbia, School of Nursing, Health Sciences Mall, Vancouver, BC V6T 1Z3 Canada; 6 The University of Melbourne, Department of Nursing, Parkville, Melbourne, Victoria 3052 Australia

**Keywords:** Prostate cancer, Partner, Football, Soccer, Exercise, Qualitative, Gender, Focus group, Older people, Dyadic coping

## Abstract

**Background:**

Prostate cancer is often labelled a couple’s disease wherein the partner plays an important role in the man’s illness management and related health promotion activities. The aim of this study was to explore partner experiences of prostate cancer patients’ engagement with a community-based football program.

**Methods:**

Eight audio-visual recorded semi-structured focus group interviews were conducted with a total of 39 female partners of men with prostate cancer who participated in a community-based football program as part of the nationwide FC Prostate Community Trial (NCT02430792). Data was managed with the software program Nvivo 11 and analysed inductively to derive thematic findings.

**Results:**

The four thematic findings were: 1) *‘Hope of a new beginning’* which included stories of hope that football would mitigate the negative effects of men’s prostate cancer treatment [s]; 2) *‘My new partner’* was characterized by attributing connections between physical activity and elevated mood as a by-product of men’s involvement in the program; 3) *‘Football first’* included assertions of the couples mutual commitment to the football program; and 4) *‘Invisible needs’* contrasted insecurity, and unforeseen challenges for partners feeling somewhat neglected. Overall, the results confirm the need for cohesion and flexibility amongst couple-dyads to ensure partners and men with prostate cancer benefit from their involvement in football programs.

**Conclusions:**

This study indicates that partners of prostate cancer survivors’ engaging with community-based football align to idealized gender relations, roles and identities. In many instances, these gendered dimensions aided positive dyadic coping and long-term exercise adherence.

**Supplementary Information:**

The online version contains supplementary material available at 10.1186/s12889-021-11448-7.

## Introduction

Prostate cancer (PCa) is the most frequently diagnosed cancer in Danish men [1]. While survival rates of localized cancer are excellent, with more than 96% of men surviving 5 years post diagnosis, prostate cancer is also the leading cause of male cancer related disability in Europe [2]. In essence, PCa treatments invoke challenges leaving many men and their partners to manage long-term side effects including altered sexual relationships. Specifically, threatened gendered identity, including sexual insecurities and role changes, constitute a central part of the distress invoked on dyadic couples affected by PCa [3].

It is evident that many men with PCa depend on their partner for emotional and practical support, and the predominant role of partners have been portrayed as “health agents and advocates” [4], “health monitors” and “selfless supporters” [5] . Moreover, female partners may experience frustration and social isolation (including feelings of being constrained/restrained in their home) when formerly active men living with PCa reduce their activity level [3, 6, 7]. Hence, interventions supporting the preservation or resumption of the couples shared activities, gender relations and roles warrants research attention.

In the context of men’s health promotion, and PCa more specifically, there have been assertions that tailored community-based PA interventions can engage men [8]. This relates to men’s preferences for active psychosocial oncology programs (as distinct from more passive talk-based modalities) and the opportunities for connecting with other men who are similarly challenged by PCa. A qualitative study confirmed that the participation in recreational football among men with PCa provided a strength-based mechanism for being responsible for their health, a practice starkly contrasting passive patient roles [9]. Participants also espoused their participation in the football program as drawing admiration from families and friends while heightening their own enthusiasm and gratitude [9]. A multicentre randomised controlled trial (i.e., The FC Prostate Community [FCPC] trial) evaluating the effectiveness of community-based football programs for men with PCa has shown much promise [10]. Specifically, the football intervention (delivered in local football clubs for one hour twice weekly) was superior to the usual care (including promotion of/referral to standard rehabilitation) in mental health outcomes after six months [11], an effect sustained after 12 months in men who continued to play football [12]. Moreover, compared to men who lived alone, the study showed that men who were living with a partner adhered more regularly to the program [12]. This finding affirmed the influence of heterosexual gender relations for promoting men’s health in the context of living with PCa [13]. Indeed, women have been socialized to care for the health of others’, especially family and the men in their lives, while men are socialized to risk rather than promote their health [14]. The male benefits of being partnered within this context have been described [9, 15]; however, the perspectives of female partners in relation to (securing) their partner’s exercise behaviour are poorly understood.

Against this background, the aim of the current study was to describe partner experiences of prostate cancer patients’ participation in a community-based football intervention. Specifically, the study addressed the research question, *What are the partner experiences of prostate cancer patients’ participation in a community-based football intervention?*

## Materials and methods

### Design

An explorative qualitative study including researcher triangulation was used. Focus group interviews were chosen to facilitate group discussions and promote interactions between participants.

### Sampling and recruitment

We purposefully sampled female partners of men with prostate cancer who had participated in a community-based football program for a minimum of six weeks as part of the concurrent FCPC trial. Partners were recruited through their FCPC playing men via mail by the lead author. Specifically, all FCPC participants who had listed a partner/spouse as contact person were informed about the study. The men were then asked to consider whether they agreed that their partner could be contacted in relation to the study. If accepted, the man provided the first name of their partner and an email-address or phone number. As such, only women whose partner had provided permission that they could be contacted received information about the study. When permission and partner contact information was available direct contact was made inviting women to take part in a focus group interview at the football club. No prior relationship was established with the women participants taking part in the study.

### Focus group

Audio and video-recorded focus group interviews were conducted by MR (female) holding a master’s degree in Public Health and appointed research assistant to the overall/parent project (i.e. the FCPC trial), and with prior experience and training in qualitative methods and PCa research. An interview guide developed by MR and JM (female, PhD, Psychologist) was used (see Additional File 1). The questions were intentionally broad and open-ended encouraging participants to share their perspectives. Notes were made by the focus group interviewer (MR) immediately after each interview; these field notes included reflections and observations in relation to the atmosphere in the room along with participants’ expressions and body language (laughter, crying, sighing, anger), informal talk and interaction among the women before and after the interview. No repeat interviews were carried out. However, emerging preliminary themes were tested in subsequent interviews. Also, a speakers’ log was kept to attach specific excerpts and narratives to the interview transcripts and contextualize quotes shared to illustrate the thematic findings.

### Analysis

All interviews were transcribed verbatim, after which the first author (JM) accuracy checked the transcriptions against the recordings (audio and visual). Transcripts were not returned for member checking to participants for comment and/or correction. Participant observation (i.e., field notes) were developed both live (i.e., during and immediately after completion of an interview) and in viewing the videotaped interviews. Braun and Clarke’s thematic analysis approach was used to jointly analyse the interview transcriptions and participant observations. The analysis was an iterative process that included the following steps:

1. *Familiarisation:* To become immersed in and familiar with the data, we (MR, JM and MK) initially individually/independently noted ideas about possible themes during the transcription, reading and re-reading of the data.
2. *Coding:* Using the software programme NVivo 11, MR systematically and independently coded the data to an agreed upon schedule using descriptive labels.
3. *Searching for themes:* MR, MK and JM each examined preliminary codes to determine themes within the data allocated to each of the codes. Some codes were subsumed, and the themes were defined and differentiated.
4. *Reviewing themes:* MK, JM and EDB reviewed initial themes together, with MK subsequently reviewing themes in NVivo 11 to check whether they worked in relation to the previously coded extracts and the entire data set.
5. *Defining and naming themes:* MK, JM and JLO collaborated to develop the analyses, refining the specifics of each theme and the overall findings to address the research question. This involved re-organising and rewriting the current manuscript to achieve coherent links between main themes and subthemes.

## Findings

Eight focus group interviews with partners of prostate cancer survivors participating in a community-based football program implemented across four football clubs in three regions of Denmark were conducted. In total, 76 invitation letters were sent to partners of FCPC players. As a result, 39 women were included in the current study and participated in the focus group interviews. In order not to exceed 10 participants per focus group, two interviews were scheduled for each of the four involved FCPC football clubs/teams. As a result, participants ranged in number from 3 to 7 for the focus group interviews. Reasons for the 37 who did not participate were no response (*n* = 13), unable to attend interview (*n* = 10), lack of interest (*n* = 10) and accepted invitation but didn’t show up to interview (*n* = 4). The average duration of interviews was 78 min (standard deviation: 15 min). The specific number of participants per interview and characteristics of their partner with prostate cancer are presented in Table 1.

**Table 1:** Number of participants and partner characteristics across interviews

	TOTAL	Club A (FG 1 and 2)	Club B (FG 3 and 4)	Club C (FG 4 and 5)	Club D (FG 7 and 8)
Number of women per interview	39	6 and 5	5 and 4	7 and 3	6 and 3
Mean age (years) of partner with PCa [min;max]	68.4 [50;80]	67.1 [54;78]	64.7 [50;75]	70 [63;80]	71.7 [68;78]
Time (months) since partner’s diagnosis of PCa [min;max]	26 [0;117]	21.3 [1;116]	35.8 [0;88]	28.7 [4;117]	18.1 [6;31]
Time (months) since partner’s inclusion in football [min;max]	14.3 [4;44]	14.2 [11;19]	11.9 [4;18]	17.6 [5;44]	13.4 [9;14]
Number of partners undergoing ADT	21 (69%)	8 (72%)	4 (44%)	5 (50%)	4 (44%)

*FG* Focus group; *PCa* Prostate cancer; *ADT* Anti-androgen therapy

Analysis of the eight focus group interviews yielded four themes and various subthemes revealing the spouses’ experiences with, and thoughts about their partner’s participation in a community-based football intervention: 1) Hope of a new beginning; 2) My new partner; 3) Football first; and 4) Invisible needs (Table 2).

**Table 2:** Overview of themes and corresponding subthemes

Main theme	Subthemes	Initial themes (Sample)
Hope of a new beginning	Powerlessness	Football as (last) resort
	In sickness and in health	Fighting back at the disease Better shape/improved fitness
	Fighting to win	Infected by partner’s happiness A new person
My new partner	Renewed strength and energy	New priority Roles and responsibilities
	Admiration and positive feedback	Affection Planning and anticipation
	Much needed sanctuary	Adjustment (of other activities) Support (keeping up motivation)
Football first	Mutual commitment	Not just for fun/a serious game Football as a male sport
	Personal sacrifices (soccer mom)	Restored masculinity Prostate cancer taboo
Invisible needs	Unacknowledged wellbeing	Anxiety and worry Changed structures of daily life
	Insecurity	Positive competition Relief of care burden
	Focus group therapy	Feeling proud

### Hope for a new beginning

The first theme, *hope for a new beginning* chronicled the challenges experienced through their partner’s PCa and the optimism afforded by the invitation to their partner to engage with a community-based football program. Fighting the disease together was described by the women as being a matter of course. They expressed the naturalness of being actively involved, explaining this as a well-established practice in their relationships. They referred to their marriage and to a long life together with their partner, which meant a bond and pledge to stick together through thick and thin. One woman explained:

“*We have been married for so many years, so it’s a natural thing that we are two; it’s not a disease he has, it’s something we need to get through together.”*

Accordingly, the women typically used the pronoun ‘we’ when they talked about their partners diagnosis, describing themselves as one half of a whole, sharing the job of managing and coping with the illness. Indeed, several participants explained precisely when *they* were diagnosed with the disease, what treatment *they* were offered and how it had affected *them*. Within the women’s narratives there was also a strong focus on understanding PCa and the disease aspects. Participant narratives typically drew from the entire illness trajectory citing the diagnosis and haste toward making treatment decisions. PSA’s, PCa staging and treatment decisions were ever present constructs that permeated the efforts (and uncertainties) of the couples. Women also sought information and affirmation from the other focus group interviewee’s with regard to their gendered practices and involvement in their partner’s illness. For example, one woman said:

“*We need to have blood tests taken in a month, and we’re already on the internet the following day to find out what the blood tests say? You do that too, right?”*

Contrasting this disease focus (and the efforts to orientate to often foreign concepts and contexts) the invitation to participate in a community-based football intervention afforded significant hope for a new beginning. Several partners talked of the football training program with deep gratitude and some talked of a strong sense of relief at the prospect of their husband being able to take part in sport with other men who were experiencing PCa. The strength-based affordances by talking to what men could do (as distinct from what they had lost through PCa and were working to recover) were paramount. Afforded was respite from the pressures that had accompanied the men’s transition to PCa and the aftermath of primary treatment [s].

*“Well, football in FC Prostate helps them not to become resigned. I mean, just give up or not motivate themselves to do anything. And in that sense, it's very important it's there. It also feels like it's part of the cure, I mean, in order to get better again you mustn't give up, right?”*

Within this context female partners talked to the value of masculine norms for being active, resilient and autonomous in fighting for their recovery. Symbolic, the will and strength to compete emerged as hope that gains were imminent despite the backdrop of PCa. Accordingly, the women described the football as a means of preventing their partner from sinking into sadness or depression wherein the intervention provided a public platform to fight back and not succumb to the disease. Timing of the invite was also critically important. The losses had indeed accumulated for many men – and by extension their partners had witnessed and experienced significant grief. In this regard, from the perspectives of partners hope for a new beginning was afforded by the football program in that it demanded the men be active, and perhaps assertive in taking up the opportunity to participate. The decision to participate was also joint in most cases, as a partner summed up in suggesting;

*“We said ‘yes’ to football right away. It came right at a time when everything was falling apart. It saved us.”*

### My new partner

The second theme, *my new partner*, highlighted the women’s experiences of the benefits that flowed from the men’s participation. Cited were physical and psychological gains for the men, and in parallel the women drew significant benefits. The transformative effects were highlighted, and several women described the feeling of having gotten their partner back as a by-product of losing the anxiety that had characterized his PCa and *their* illness journey, *“.. then he got started on this football and he revived completely and became a completely different person again.”*

The women appreciated the men’s familiarity with football, one that deeply aligned to pleasure and lifelong culturally and gendered normed interests. This in turn signaled a break from the biomedical focus on PCa and offered hope that perhaps things previously enjoyed (such as football) might be re-instated:

*“It’s wonderful to see that he has found his playful inner child; it’s really good, he puts his life and soul into it!”*

With pride (admiration) and joy in their voices the women described experiencing a positive change in their partner’s physical prowess including improved ability to complete daily tasks, which they attributed to the conditioning that football provided. The physical benefits transcended the domestic sphere wherein men became more active in the home expending their newfound energy to benefit the couple:

*“I can tell that his fitness has significantly improved. We live on the third floor and now he’s the one carrying our groceries from the ground floor to our apartment.”*

The sense of purpose and resumption of preexisting gender roles increased the presence of the men, simultaneously easing the work (and worry) of many female partners. Some women described how the football had given them the opportunity to actively affirm their new partner efforts towards better health and physical activity in their everyday lives. For some, football also levered their caregiving capacity through collaborative efforts for joint fitness. These gendered practices often included some friendly rivalry, as illustrated by these quotes:

*“It really has brought a change to our lives, a change for the better! We have a two-year-old grandchild and he has so much more energy to spend time together with him.”*

*“It’s grown out of the football, I’m pretty sure that’s what set us off and got us going with doing a physical activity together.”*

*“All the family join in [annual family run] and this year he joined in and he ended up beating me, so his fitness has definitely improved.”*

The women affirmed each other, collectively nodding in assent, that there had also been psychological changes in their men. They smiled and laughed explaining that the positive change in their partner’s moods probably had something to do with feeling stronger and being able to manage more physically. Here the relational aspects emerged wherein the partner talked to the muscularity embodied by her husband – as a source of self-esteem that flowed benefits to his mood. A participant exclaimed:

*“He can feel his muscles are better and he has it on paper, too, that they have become, yes bigger or better, so he’s happier in one way or another and that’s a strange thing, right? I actually think I can feel that as a wife.”*

According to the women, after training their partners talked keenly and earnestly about what occurred, which for the participants suggested enthusiasm and commitment, ‘things’ that had been absent in the presence of PCa. A woman explained:

*” My team lost today! Never mind, I said, isn't it all just a bit of fun? ‘No way! It’s deadly serious – we can’t lose!’ There would have to be a major reason for him not to go to the training.”*

The women reported that their partners were proud to contribute, which they put down to football and the camaraderie with the other men that came from being in a team and competing with others. The women referred to the football as real “men’s sport”, which their partner felt comfortable with because it was a familiar activity. The women agreed that football supported their partner’s feeling of being a man and that it relegated PCa to the background:

*“It's a male activity taking place in the middle of the treatment for the illness and all the things about it [PCa] that make you feel somehow unmasculine.”*

Indeed, many of the men were retired, and the football commitment offered purpose, social connection and some legitimate time away from home – as well as respite from the uncertainty that could accompany PCa and its treatments. The women described how football had provided a kind of sanctuary, and how they were reassured that their partners were renewed and regaining some vigour through being involved. A woman explained:

*“I also think I can notice the effect of the social aspect on my husband, the fact that they talk together, although they don't all have the same diagnosis or have had surgery for the same thing or to the same degree, but I can tell from my husband’s reaction that he really enjoys himself.”*

### Football first

The third theme related to the women’ accounts of the couple’s mutual commitment to the men’s involvement with the football program, including experiences of how football had become a priority overriding other non-football activities. When asked about the importance of the football training for managing the disease, the women answered again in the third person plural, affirming, “*We’ve become very fond of it [football]*)” explaining that the football provided a new positive and shared focal point in their joint lives. Another woman quipped:

*“There’s nothing above it [football]. In America, they say America first, but at our house it’s prostate football first.”*

The women recounted in appreciative terms that their partner was very persistent when it came to attendance at football practice, as one woman said, “*I don’t think he has ever missed football practice, not even once.”*, a position affirmed by another participant:

*“He wouldn’t dream of not going [to football]. Whatever else might be planned for that day, it just has to be cancelled, it can’t get in the way of him playing football!”*

The women described how they were actively involved in planning for their partner’s football training. Many of them talked of how football occupied their lives and the rituals that were revisited in the pre-game and practice habits. One woman explained:

*“His clothes and gear are organised in the evening. I tell you, they are lined up on the dining room table. And when it’s time to leave, he’s like a small boy who can’t wait.”*

The women also described how they were careful to offer support when bad weather or other obstacles challenged to their partner’s motivation, which, the women suggested, was more the case at the start of the intervention (i.e., after a short period of participation).

*“Once it was raining and he stood there looking out of the window … So I said, ‘pack your things and just go over there, if you're the only one you'll be sent home again!’ So he went and he found 10 other guys were there! Since then he’s gone there every single time.”*

The women recognized the importance of the football in their partners’ lives. They do not complain, and for the most part selflessly compromised to sustain their partner’s involvement with the program. One woman explained:

*“We need a car to go everywhere and if he’s [husband] off to football, I have to adjust accordingly.”*

Most participants expressed a strong interest and desire to be a part of their partner’s football practice in the same way as they take part in the treatment. One woman described how she once watched her husband and his team during football practice without her husband knowing, which made the other women in the interview laugh, and ask her questions about what she saw. As such, the women shared a curiosity about what goes on among the men, but at the same time described respecting and appreciating the fact that the football was exclusively for the men and a part of their partner’s therapy that she was not part of.

*“It’s okay that it’s [football] something just for them – something that it theirs only.”“Exactly, I think so to.”*

*“Maybe they wouldn’t feel as emancipated if we were there with them.”*

*“We would probably have drowned them with our talk. There’s no way of denying that we [women] talk a lot” [laughter].*

### Invisible needs

The fourth and final theme, *invisible needs*, related to some women’ feelings of insecurity, and the lack of recognition and appreciation for their support. Often, the women shared detailed accounts of the time around diagnosis and asked each other about treatment side effects in ways that were unrelated to the football program. One woman brought along a small black notebook in which she had written down questions/subjects she would like to discuss with the group:

*“But I would like to talk a bit about how my husband has reacted, maybe you’ve experienced the same? … I’d like to talk a bit more about the psychological side of things because I’ve not had the chance to talk about it with others and hear if they’ve had the same experience"*

The interviews were characterized by a willingness of the women to admit vulnerability and fragility within and without their intimate partner relationships, and a preparedness to share perspectives and comfort one another. Several participants got emotional during the interview and were supported by the others around the table. For example, responding to a participant’s disclosure about feeling isolated, one woman said:

*“It sounds like you’ve had a terrible experience dealing with the people who are supposed to take care of you”* and *“I really feel like giving you a hug.”*

The women recounted that they did their utmost to be supportive, stay calm and to be strong. It was important for them to give the impression of being resilient so that neither their partner nor their children worried. Two participants explained;

*“I do think there are a lot of things you have to just swallow one way or another.”*

*“That you’re holding back?”*

*“Yes, I think so, I do that to … ”*

*“ … not to hurt him?”*

*“Yes.”*

However, the women explained that their resilience and stamina meant that friends and family were unaware of the strain and burden the illness brought to bear. In their efforts to put on a brave face, some women explained feeling lonely and exhausted;

*“It can be depressing as caregivers to be positive and cheerful all the time.”*

The partner’s participation in the football program meant acknowledging and being open about the PCa, which was a challenge for some women who wished to be discreet and private about the disease, purposefully limiting how many people in their social circles knew about it:

*“I think it’s been tough. Not because I’m like that [weak]. I actually think I usually have quite a lot of strength to handle things. But my husband feels like nothing should be swept under the carpet, so he called everyone we know and explained that he was sick and what the treatment was. I was at my limit and about to say something to him straight out because I heard him explain the same thing a 100 times in one night and I thought: I need a break now. And then people called and came round, and I thought I simply can’t take it anymore. But he needed it, so I thought I should keep my mouth shut. All I was thinking was I want to escape.”*

The women did not complain about the attention that their partner received because of his participation in the football program; yet they described their invisibility in the ‘couple’s disease’:

*“I remember everyone coming and asking, ‘How are you?’ to my husband. You never ask the spouse how they are. There are so few people who ask how* you *are doing and when they do ask, woooshh, all the tears come pouring out because no one really ever asks about it; it’s always the person who is ill who they ask about. I think that can be tough sometimes.”*

However, when discussing the need for spouse support and if a program tailored for partners would be helpful (i.e., similar to the football program for the men), the women were reticent to be involved. For example, one woman said, *‘No, that’s not for me”; “I probably couldn’t fit it into my calender*”, while another suggested, *“I don’t think too much about that [need for support]”.* Nevertheless, the women often affirmed each other in sharing their experiences, and at the end of the interviews the women said that it had been nice to chat, and in several cases, they took the initiative to continue the conversation after the interviews had finished:

*“We can talk for a long time! Because of course this has taken up a lot and still takes up a lot somewhere in our lives, right?”*

*“It’s been good for us to talk a bit.”*

While many partner needs were invisible there was also a commitment to maintaining that status quo for many women. Herein the tensions for selflessly supporting their men emerged, amid disclosures about the fatiguing hard work that flowed from their efforts to be strong for others.

## Discussion

The current study explored the experiences of female partners of men with PCa in relation to their partner’s participation in community-based football. Revealed was how men’s participation can revitalize and re-imagine couple-dyad coping through mutual commitment and restorative gender relations, roles and identities. In this sense, it might be reasonably argued that football provided a vital distracter from PCa disease and explicit avenues for illness management. Of course, there were unintended effects for partners as well, wherein there were experiences that suggested partner invisibility could grow in step with the men’s over involvement with football. On balance, however, it was clear that the women could see, and fully endorsed their men’s involvement with football as a much needed tonic in their fight against PCa. In what follows we discuss the themes developed through our analyses with a view to augmenting the existing literature and pointing toward future avenues for PCa research interventions.

Results of this study confirm the centrality of intimate relationships in PCa, and the need to garner hope for men and their partners [4, 16]. Herein the invitation to play football was understood as offering tangible opportunities to counter what might emerge as ever oppressing dire and challenging illness issues. According to the participants, the decision to take part in the football intervention was made in the hope that it would be therapeutic, help to boost energy levels and [re] create a fighting spirit during a time characterized by vulnerability and fatigue. Some women described significant caregiver fatigue when their men joined the football intervention. Indeed, the women’s accounts of their partner’s participation in the football intervention suggested that the women appreciated the program because it provided a public platform for their partner to express and experience strength, competitiveness, comradery and pride. Afforded were their normative masculine practices at a time when other masculine ideals were threatened or lost (i.e., erectile function, incontinence). The women’s perspectives revealed sophisticated relational understandings that the normative masculine gains inherent to playing football could buoy their men. By extension, the participant’s caregiver fatigue could be (and often was) reduced. That said, as a couples’ disease, many participants were also involved as (potentially invisible, unrecognized) background supporters encouraging and applauding men’s involvement. Bottorff et al. [16] reported similar gendered dimensions in the context of men’s involvement with PCa support groups. Likewise Oliffe et al. [17] suggested that dominant ideals of femininity could extend and retract within and across the public and domestic spheres where couples engage PCa related issues. The results also confirm previous qualitative work describing how men with PCa who take part in a football intervention appreciate being able to share new stories from training and the value adding aspects for their husband and father operating predominately in the domestic sphere [9, 15]. Moreover, the results confirm the findings of a recent meta-synthesis of various exercise interventions and their effect on male perception of masculinity, body image, and personal identity, which concluded that exercise can facilitate a process of self-reflection secondary to changes in physique and help to re-establish male self-efficacy [18, 19] .

Gender is of course relational, and couples’ response to PCa is part of an interactional system [20]. In the context of supporting a prescribed football intervention, the women included in this study understood the draw and value for their men to participate. Of importance, the women in the current study appeared to acknowledge the private and public elements of masculinities and how men crave both. As such, the results of the current study confirm the potential benefits of capitalizing on the cultural representation of football as a sport that builds on and cultivates co-constructed masculine ideals [15]. As such, the findings align with a recent study of the effects of couple-focused interventions for men with localized PCa and their spouses, which suggested that endorsement of traditional masculine norms may be requisite for improving psychological and relationship function [21].

Also inherent was the recognition that men can benefit by being with other men to discuss vulnerabilities and uncertainties, potentially alleviating women’s aloneness in their caregiver roles. In essence, while women could assure men of their masculinity, the affirmation and normative frames of being in the company of men who experience PCa was also recognized as uniquely important by the women. Another notable finding of the current study relates to the the nurturing of the women for one another, and the role that the women described occupying within the relationship when the partner was involved in football, reflecting feminine ideals of caring and compassion previously described in a study of women’s motivations and psychosocial benefits in relation to attending PCa support groups [16]. The processes witnessed among the women in the focus group interviews reflect normative frames of femininities (i.e., caring, nurture, collaboration, touch, talk) that are in contrast to the men who, as described in previous related studies [9, 15], use normative masculinities (i.e., competitiveness, physical prowess, resilience) to connect with one another and perhaps themselves as men.

Although the football intervention, according to the women, appeared to have re-installed a sense and demonstration of self-reliance and independence in their partner, the women still portrayed themselves as health supporters and cancer co-survivor including a willingness to set aside personal activities confirming their role and tasks in relation to encouraging health behaviour and exercise adherence [22, 23]. Consequently, future work might benefit by exploring the potentially compromising role of football on health and psychosocial needs as previous described in the scientific literature on informal cancer care [24–26]. Also, it is clear from the current study that the football did not directly remedy women’s supportive care needs. As previously stated, the focus group moderator noted the women’s eagerness and need to discuss and share their PCa experiences, in part as a means to reducing the isolating effects of their experiences. These discussions often back grounded the football program, even though that was the focus on the interview.

Thus, while benefits of football for the man with PCa and his partner (especially related to the man’s response to football) were evident, football may not directly aid the well-being of all female partners.

Hence, while previous studies point to the relevance of couple-based interventions and/or women’s involvement in PCa support groups, the current study findings suggest that women may benefit from opportunities to socialize with each other without the presence of their partner. Interestingly, however, when asked directly about their interest in programs specifically developed for partners, the women expressed little interest in formalizing such connections. Thus, while it is tempting to suggest that talk based interventions might suffice by trading on normative femininities to connect women partners there may also be important gender transformative opportunities. For example, the emergence of elite female athletes and teams (including soccer) have transformed idealized femininities to include physical prowess and competitiveness. In the context of female partners, it might well be that a women’s football league could also satiate (at least in part) their often neglected needs. Accordingly, more research is warranted to examine the role of spouses, and the effects of partners’ support in relation to exercise-based cancer rehabilitation sport programs including the FC Prostate Football initiative or dragon boating teams exclusively for survivors of breast cancer [27].

### Methodological considerations

Despite numerous previous studies documenting the benefits of having a partner in relation to PCa survival, and studies showing benefits of regular exercise in men with PCa, the current study is, to our knowledge, the first study to examine the perspectives of PCa partners in relation to their husband’s participation in an exercise intervention. The study provided rich data to a specific (i.e., narrow) question in a somewhat homogenous population. Data saturation was achieved within the inductive analyses, evidenced by the strong agreement and representation across the four themes. Moreover, credibility of study findings was supported via inclusion of participants from different regions/parts of the country (including more rural areas) reflecting potential differences in cultural values. Confirmability was ensured via the involvement of a multidisciplinary team of researchers involved in analysis and interpretation (i.e., researcher triangulation). Specifically, audio-visual recordings of the interviews facilitated multiple researchers being exposed to the data collection contexts and the interpretations made. Furthermore, reliability was secured via use of both video and audio recordings, and verification of the transcriptions’ accuracy.

There are, however, also several study limitations. First and foremost, the transferability of the study findings are limited. While it may be argued that any team-based sporting activity involving exercise and camaraderie would produce similar results, the current study was carried out within the rich cultural contexts that embrace and celebrate football in Europe. Moreover, the partner relationships, role and identities might also be understood as reflecting cultural and temporal touchpoints unique to the study setting. Also, the fact that we included only women whose men were motivated to join football (i.e., FCPC players), and whose men agreed that she could be contacted by the research team (i.e., patient-husband functioned as gatekeeper to their partner’s involvement in the study), imply that only the voice of couples characterized by an interest in football or an interest in and ability to mutually share information has been communicated. Hence, generalizability to other PCa men and their partners is neither the aim nor claim of the current research. Likewise, the findings cannot be assumed to fit other cancer types. Moreover, of the 76 women approached, only 39 (51%) were included. This modest response rate and the lack of access to information about partners who did not respond to invitation to participate leaves us with some uncertainty as to whether the women included may be the ones who were especially positive about their partner’s involvement in football. Finally, although interviews were filmed and indicated vibrant interaction among participants, only one interviewer (moderator) was present during the interviews providing limited field notes to depict and analyse the group dynamics *during* interviews.

## Conclusion

In conclusion, this study suggests that among partners of men with PCa, the patient-husband’s participation in football may facilitate mutual commitment and restored gender roles although spouses’ needs should also be explicitly addressed. Future team sport and exercise training interventions may benefit from capitalizing on partners’ supportive role while also incorporating potentially unmet support needs of partners. Additional studies examining the impact of gender sensitized and/or diagnosis specific exercise-based rehabilitation on well-being of partners of cancer survivors are warranted.

## Supplementary Information

**Additional file 1.** Interview guide

## Data Availability

Due to privacy protection issues and according to Danish legislation, the datasets supporting the conclusions of this article are available only to the researchers involved in the project. However, after the principal researcher (JM) of the FCPC project consults with the review board, data may be available upon reasonable request.

## Abbreviations

PCa: prostate cancer
FCPC: FC Prostate Community
