# Supplementary material for: Female partner experiences of prostate cancer patients’ engagement with a community-based football intervention: a qualitative study
Source: BMC Public Health. 2021 Jul 15;21:1398. doi: 10.1186/s12889-021-11448-7 (PMC8281704; doi:10.1186/s12889-021-11448-7)
Supplement: Supplementary file 1 — Additional file 1. Interview guide [file 12889_2021_11448_MOESM1_ESM.docx]

**Additional File 1.**

**Interview guide**

**Intro***Introducing myself*
Mette Rørth (Master of Public Health Studies, appointed research assistant, previously studied impact of injuries)

*Introducing the study*

In the FC Prostate Project, as you may know, we want to investigate whether recreational football can retain men with prostate cancer to be physically active and improve their quality of life, muscle mass and bone strength. But beyond that, from the start, it has been part of the project to explore how partners to the participants may be affected by their husband’s participation. Prostate cancer is sometimes labelled a "couple's disease", and several studies have shown that prostate cancer in particular affects relationships and spouses. The purpose of this interview is to hear your experiences, thoughts and attitudes towards the project and your reactions to each other's experiences. It might sound a little more ‘solemn’ or strict than it really is. In fact, it is "just" a loosely structured talk between you, where I am in on a listener. I have designed a so-called interview guide, which we should in no way follow slavishly, but which may support the conversation. I know that for some it can be a sensitive topic to talk about this, so we just take it quite slow and if you don't want to say something it's obviously okay. No one will be directly asked anything. Furthermore, it is important to say that if you need to leave, you just get up and sneak out the door. The interview here, together with seven other interviews of the same kind, will be included in one or more articles in which they, the interviews, have been analyzed and interpreted. Before we start, do I need to make sure it's ok for me to record video and audio? And in doing so, I also need you to sign a consent form.

**Themes**

*Partner’s enrolment in football*

When you heard that your partner was going to start football, what were your thoughts/reflections?

*Partner’s adherence to football*

What is your opinion about your partner playing in FC Prostate?

Do you talk about football at home? Does your partner talk to you about what the team does and gets up to at training?

What difference, if any, do you think FC Prostate has made for your partner?

What difference has the football made for you?

*Impact of football on coping with prostate cancer*

Do you think that the football has had any kind of effect on how your partner has managed his prostate cancer? How?

Has the football influenced how you manage your partner’s prostate cancer? How?

Do you think that the football has had any effect on your intimate relationship? Has it influenced your intimacy?

*Perceived changes in physical function*

Do you notice any physical changes in your partner? Has he become stronger or more mobile, or does he give the impression of being fragile and sore most of the time?

Is there anything you can do now that you could not do before or anything the football stops you from doing?

How do you think your partner physically benefits from playing football?

*Perceived changes in psychosocial function*

Do you notice any physical changes in your partner? Less or more energy? Enjoyment from the game, the social contact? New relationships? Old relationships?

In your view, how can the men benefit each other on the team?

*Support needs of female partners*

Is there anything you as a family member feel is missing? Have you been offered the help and support you have needed during your partner’s illness? More specifically, do you have any requests or ideas for support that could be offered to you as next of kin?

**Outro**

Thank you very much for your kind participation. It’s been a privilege to talk to you and your experiences are of great value and will help extend/advance our knowledge in this field. You’re welcome to stay in the room if you wish. You are also welcome to get in touch/reach out if there is anything that you feel that you need to add or that you wish to change/withdraw.
